# Cognitive function in toddlers with congenital heart disease: The impact of a stimulating home environment

**DOI:** 10.1111/infa.12376

**Published:** 2020-11-19

**Authors:** Alexandra F. Bonthrone, Andrew Chew, Christopher J. Kelly, Leeza Almedom, John Simpson, Suresh Victor, A. David Edwards, Mary A. Rutherford, Chiara Nosarti, Serena J. Counsell

**Affiliations:** ^1^ Centre for the Developing Brain School of Biomedical Engineering and Imaging Sciences King’s College London London UK; ^2^ Paediatric Cardiology Department Evelina London Children’s Healthcare London UK; ^3^ Department of Child and Adolescent Psychiatry Institute of Psychiatry, Psychology and Neuroscience King's College London London UK

**Keywords:** cognitive development, congenital heart disease, stimulating parenting

## Abstract

Infants born with congenital heart disease (CHD) are at increased risk of neurodevelopmental difficulties in childhood. The extent to which perioperative factors, cardiac physiology, brain injury severity, socioeconomic status, and home environment influence early neurodevelopment is not clear. Sixty‐nine newborns with CHD were recruited from St Thomas’ Hospital. Infants underwent presurgical magnetic resonance imaging on a 3‐Tesla scanner situated on the neonatal unit. At 22 months, children completed the Bayley Scales of Infant and Toddler Development‐3rd edition and parents completed the cognitively stimulating parenting scale to assess cognitive stimulation at home. Level of maternal education and total annual household income were also collected. Hospital records were reviewed to calculate days on the intensive care unit post‐surgery, time on bypass during surgery, and days to corrective or definitive palliative surgical intervention. In the final analysis of 56 infants, higher scores on the cognitively stimulating parenting scale were associated with higher cognitive scores at age 22 months, correcting for gestational age at birth, sex, and maternal education. There were no relationships between outcome scores and clinical factors; socioeconomic status; or brain injury severity. Supporting parents to provide a stimulating home environment for children may promote cognitive development in this high‐risk population.

## 1 INTRODUCTION

Congenital heart disease (CHD) is the most common congenital abnormality, affecting approximately 1% of neonates (EUROCAT, 2015; Hoffman & Kaplan, 2002). Advances in clinical care mean increasing numbers of infants with CHD are surviving into adulthood (Wren & Sullivan, 2001), yet survivors are at increased risk of neurodevelopmental disorders (Latal, 2016). Up to 50% of children with CHD show developmental impairments including mild intellectual difficulties, poor motor skills, attention and executive function impairments, and speech and language disorders (Gaynor et al., 2015; Gerstle et al., 2016; Mebius et al., 2017; Sarrechia et al., 2016; Wray, 2006). As survival has increased, the focus of research has shifted to understanding factors associated with improved cognitive and behavioral development in this population (Marino et al., 2012).

Previous research has investigated the impact of disease‐related and perioperative clinical factors on outcome. Early term birth (<39 weeks) (Costello et al., 2010; Gunn et al., 2016), increased time to surgical repair of heart defect (Anderson et al., 2015), presence of brain injury (Claessens et al., 2018; Mebius et al., 2017), cyanotic heart disease (Wray & Sensky, 2001), and length of hospital stay post‐surgery (Gaynor et al., 2014, 2016; Gunn et al., 2016; Hansen et al., 2016) have been implicated in adverse clinical and cognitive outcomes in children with CHD; however, results are inconsistent between studies.

In addition to clinical variables, the impact of environmental factors on development is of interest in this population (Miguel et al., 2019). Family environment and socioeconomic factors such as deprivation and maternal education impact cognitive and executive function development in healthy children (Bradley & Corwyn, 2002; Shah et al., 2016; Zhou et al., 2007) and pediatric clinical populations (Bradley et al., 1994; Coscia et al., 2001; Downes et al., 2019; Linver et al., 2002; Treyvaud et al., 2012). Lower maternal education has been associated with poorer cognitive outcome in infants with CHD (Gaynor et al., 2015). To our knowledge, there are no studies assessing the relationship between home environment and cognitive development in children with CHD.

The aim of this study was to investigate whether perioperative clinical factors, severity of brain injury, type of CHD, stimulating home environment, and socioeconomic status predict cognitive, language, and motor abilities at 22 months in children with CHD.

## 2 METHODS

A prospective cohort of 69 infants with critical or serious CHD were recruited after birth from the Neonatal Unit at St Thomas’ Hospital, London between 2014 and 2017. Critical CHD was defined as hypoplastic left heart syndrome, transposition of the great arteries, pulmonary atresia with intact ventricular septum, interruption of the aortic arch, and all infants requiring surgery within the first 28 days of life with the following conditions: coarctation of the aorta; aortic valve stenosis; pulmonary valve stenosis; tetralogy of Fallot; pulmonary atresia with ventricular septal defect; and total anomalous pulmonary venous connection. Serious CHD was defined as any cardiac lesion not defined as critical, which requires cardiac catheterization or surgery before age one (Ewer et al., 2011; Kelly et al., 2019). Exclusion criteria included suspected or confirmed chromosomal abnormality or congenital syndrome, previous neonatal surgery before recruitment (excluding cardiac catheterization procedures) or suspected congenital infection (Kelly et al., 2019).

The project was approved by the National Research Ethics Service West London committee (07/H0707/105), and informed written parental consent was obtained during the neonatal period and at follow‐up in accordance with the declaration of Helsinki.

Hospital records were reviewed to calculate days on the intensive care unit (ICU) post‐surgery, time on bypass during surgery, and days to corrective or final palliative surgery. In children who underwent more than one surgery, days on ICU and time on bypass were summed across procedures.

### 2.1 Magnetic resonance imaging

Infants underwent magnetic resonance imaging (MRI) during the neonatal period before cardiac surgery. Neuroimaging was performed on a 3‐Tesla Philips Achieva MRI system situated on the Neonatal Unit at St Thomas’ Hospital with a 32‐channel head coil. T1‐weighted magnetization prepared rapid acquisition gradient echo (repetition time (TR): 17 ms, echo time (TE): 4.6 ms, inversion time (TI): 1465 ms, flip angle: 13°, resolution: 0.8 × 0.8 × 0.5 mm^3^ or TR: 11 ms, TE: 4.6 ms, TI: 714 ms, flip angle: 9°, resolution: 0.76 × 0.76 × 0.8 mm^3^), T2‐weighted fast spin echo (TR: 14,473 ms, TE: 160 ms, resolution: 0.86 × 0.86 × 1 mm^3^, or TR: 12,000 ms, TE: 156 ms, resolution: 0.8 × 0.8 × 0.8 mm^3^), susceptibility‐weighted MRI (TR: 32 ms, TE: 25 ms, flip angle: 12°, resolution: 0.4 × 0.4 × 1.8 mm^3^), and diffusion‐weighted MRI (TR: 7536 ms, TE: 49 ms, resolution: 1.75 × 1.75 × 2 mm^3^, 32 non‐collinear directions, b‐value; 750 s/mm^2^ or TR: 3800 ms, TE: 90 ms, resolution: 1.75 × 1.75 × 1.5 mm^3^, 300 non‐collinear directions, b‐values: 400, 1000, 2600 s/mm^2^) were collected. MRI was performed during natural sleep without sedation, and pulse oximetry, respiration, temperature, and electrocardiography were monitored throughout by a nurse and pediatrician experienced in neonatal MRI procedures.

Images were reported by two neonatal neuroradiologists. All images were subsequently rereviewed to ensure consistency, and lesions classified as focal arterial ischemic stroke, white matter injury (WMI), cerebellar hemorrhage, or intraventricular hemorrhage as we have reported previously (Kelly et al., 2019). The location and properties of lesions on T1‐weighted and T2‐weighted imaging, susceptibility‐weighted imaging and apparent diffusion coefficient maps were recorded. WMI was classified into normal (no WMI), mild (≤3 foci and all ≤2 mm), moderate (>3 and ≤10 foci or any >2 mm), or severe (>10 foci) (Beca et al., 2013).

**Figure 1:**
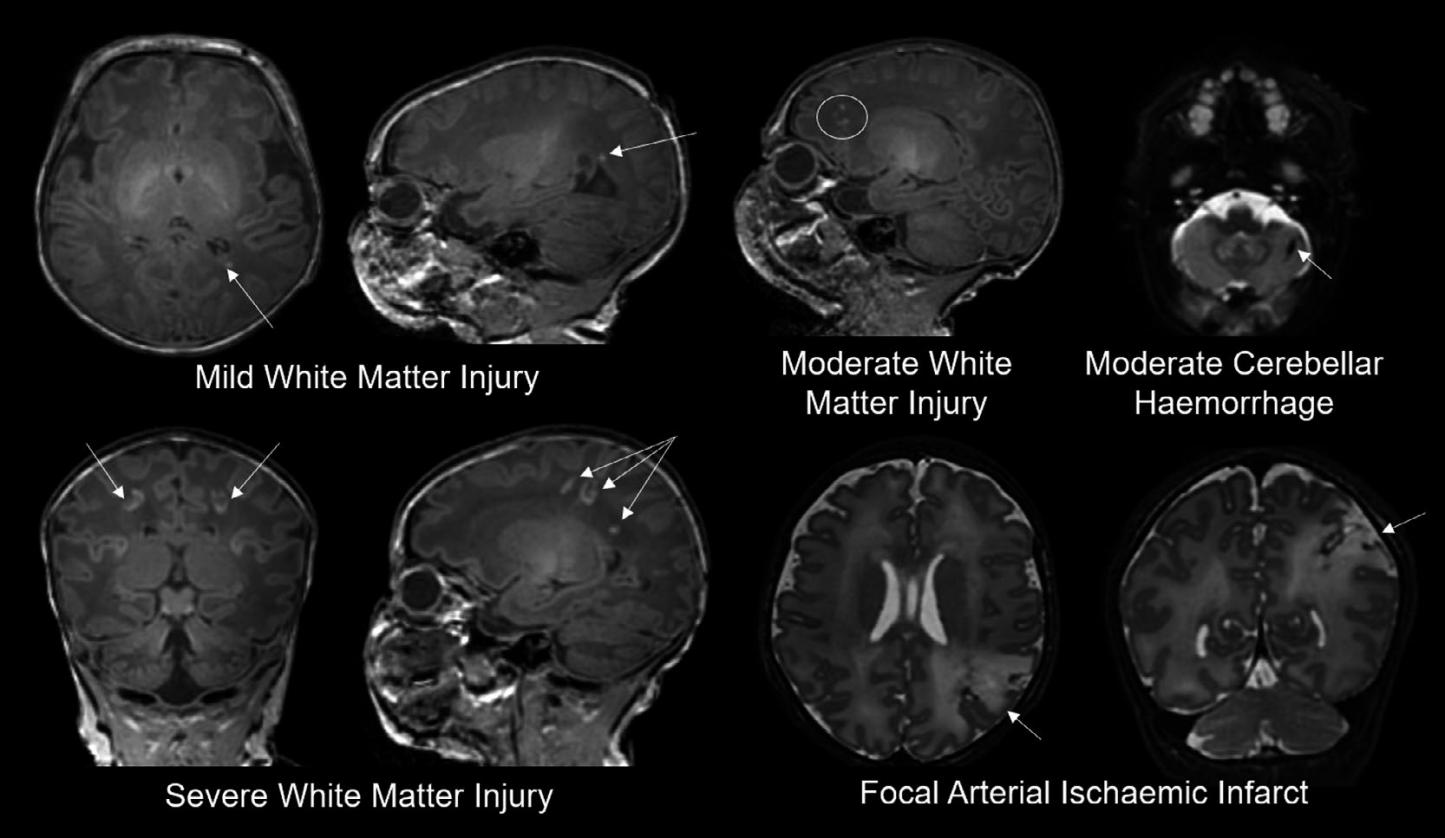
Examples of mild, moderate, and severe brain injury identified in the cohort

Overall each baby was categorized into one of four brain injury groups: normal, mild (intraventricular hemorrhage, and/or cerebellar hemorrhage ≤2 mm, and/or mild WMI), moderate (cerebellar hemorrhage >2 mm and/or moderate WMI), and severe brain injury (focal arterial ischemic stroke and/or severe WMI) (see Figure 1 for examples of injuries) (Kelly et al., 2019).

### 2.2 Follow‐up assessment

Sixty‐four infants (five died before follow‐up) were invited to attend a follow‐up assessment at 22 months either at St Thomas’ Hospital or as a home visit. Sixty‐two infants (two refused follow‐up) were assessed at 22 months by a developmental pediatrician (AC) blinded to cardiac diagnosis and clinical factors. The Bayley Scales of Infant and Toddler Development‐3rd edition (Bayley‐III) was administered to obtain standardized cognitive composite scores, language composite scores, and motor composite scores (Bayley, 2006).

The cognitively stimulating parenting scale (Wolke et al., 2013) was completed by parents during the follow‐up assessment. This is a 21‐item questionnaire adaptation of the Home Observation for Measurement of the Environment (HOME) Inventory (Bradley & Caldwell, 1984) designed to assess the level of cognitive stimulation available in the home (acceptable internal consistency: Cronbach α = 0.77). Sixteen items are yes/no response questions investigating the child's access to stimuli (six questions; in your home, does your child have access to: e.g., toys that teach colors and shapes?); parental interactions (seven questions; in your home, do you teach your child: e.g., animals’ names?), and parental behaviors (three questions: e.g., do parents read in their free time?). Five items are scales investigating frequency of reading/telling stories between parents and infants, number of books in the home, frequency of family excursions, frequency of family holidays, and frequency of museum visits. The original yes/no response questions were adapted to reflect current technological advances: one question was changed from does your child have access to a “cassette player” to “cassette/CD/DVD player,” and three questions were added asking the following: does your child have access to “YouTube,” “computers/iPads/iPhone,” and “learning apps such as Peak‐a‐boo, Peppa Pig or Fish School.” Four additional questions were added to the yes/no scale: does your child have access to gross motor toys such as trains cars bikes to sit on and push along?; does your child have access to a child‐size table?; does your child have access to toys that teach household tasks such as sweeping, ironing, washing or other daily activities? and do parents follow current affairs? For the 23 yes/no items, “yes” was scored as “1” and no was scored as “0.” Responses were totaled across the yes/no items and scale items to give a cognitively stimulating parenting scale score (minimum 0, maximum 46).

Parents were also asked to report total annual household income (eight categories: <£20,000, £20,000–29,999, £30,000–39,999, £40,000–59,999, £60,000–79,999, £80,000–99,999, £100,000–149,999, >£149,999) as a measure of socioeconomic status and maternal education level (four categories: GCSE/O‐levels, A‐levels, vocational/college education, higher education) at time of follow‐up assessment.

### 2.3 Statistical analysis

Histograms and skewness and kurtosis values were used to assess normality. Bayley‐III scores were compared to the standardized test mean (100) using one‐sample t tests. Associations between categorical variables and Bayley‐III scores were tested using Kruskal–Wallis H and Mann–Whitney *U* tests. Associations between continuous variables and Bayley‐III scores were tested using Spearman's rank correlations. Significant variables were entered into a multiple linear regression to predict Bayley‐III scores co‐varying for sex, gestational age at birth and maternal education level (GCSEs/O‐levels as the baseline level of education). Missing data were excluded from analyses in a pair‐wise manner. Holm correction was used to control the family‐wise error rate for multiple comparisons (corrected *p*‐values reported as pFWE). Groups with three or fewer participants were combined with adjacent groups for statistical analyses. When comparing household income levels, responses were combined into four categories (<£30,000, £30,000–59,999, £60,000–99,999, >£100,000) to ensure adequate statistical power. Statistical analyses were performed in SPSS version 24 and Python version 3.7.

## 3 RESULTS

**Figure 2:**
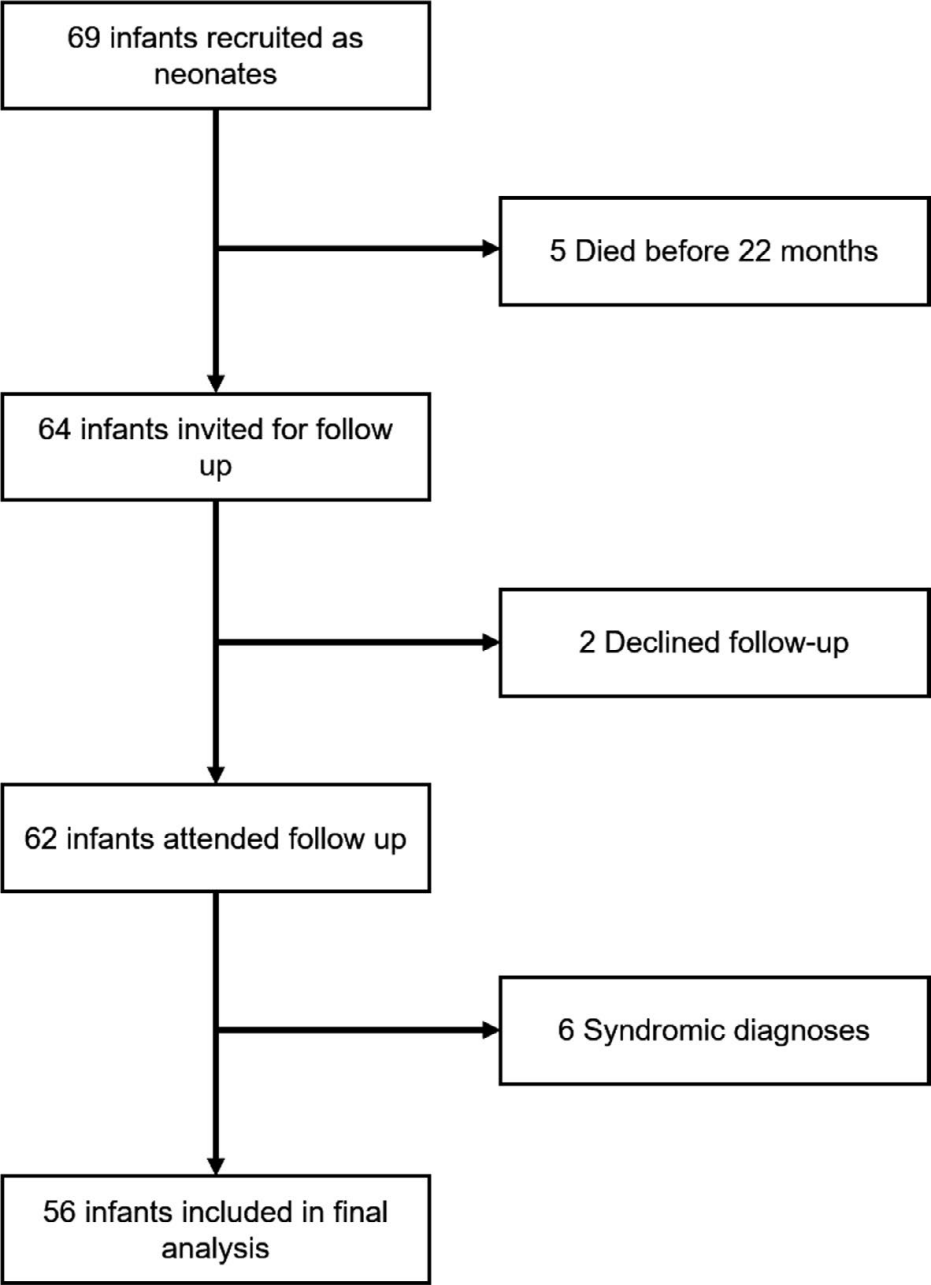
Flowchart of participant recruitment from initial consent to final analysis sample

The final analysis sample included 56 infants (six with syndromic conditions diagnosed after recruitment were excluded; Figure 2) with heterogeneous diagnoses of CHD who underwent follow‐up assessment at a median of 22.23 months (Table 1).

**Table 1:** Characteristics of the final analytical sample of children with CHD

Measure	*N* = 56
Demographics	
Age at birth median (IQR)	38.43 (0.57)
Corrected age at follow‐up in months median (IQR)	22.23 (0.722)
Male number (%)	31 (55.4)
Cardiac physiology	
Cyanotic heart disease number (%)	44 (78.6)
Heart defect causing abnormal mixing of blood	
Transposition of the great arteries number (%)	26 (46.4)
Truncus arteriosus number (%)	2 (3.6)
Left‐sided abnormalities of the heart	
Coarctation of the aorta number (%)	9 (16.1)
Hypoplastic left heart syndrome number (%)	2 (3.6)
Right‐sided abnormalities of the heart	
Tetralogy of Fallot number (%)	9 (16.1)
Pulmonary stenosis number (%)	3 (5.4)
Pulmonary atresia number (%)	3 (5.4)
Tricuspid atresia number (%)	2 (3.6)
Clinical characteristics	
Brain injury seen on presurgical MRI number (%)	
None	35 (63.6)
Mild	11 (20)
Moderate	6 (10.9)
Severe	3 (5.5)
Cumulative number of days on ICU post‐surgery median (IQR)	4.5 (4.75)
Days to corrective or palliative surgery median (IQR)	16.5 (140.5)
Cumulative minutes on bypass during surgery median (IQR)	149 (113.75)
Follow‐up assessment scores	
Bayley‐III cognitive composite score mean (SD)	91.5 (13.3)
Bayley‐III language composite score mean (SD)	89.8 (16.6)
Bayley‐III motor composite score mean (SD)	93.4 (12.8)
Cognitively stimulating parenting scale mean (SD)	31.1 (6.73)
Socioeconomic factors	
Highest maternal level of education at follow‐up assessment number (%)	
GCSE/O‐levels	6 (12.5)
A‐levels	4 (8.3)
Vocational/college education	13 (27.1)
Higher education	25 (52.1)
Annual household income at follow‐up assessment number (%)	
<£20,000	7 (12.3)
£20,000–29,999	4 (7)
£30,000–39,999	6 (10.5)
£40,000–59,999	11 (19.3)
£60,000–79,999	4 (7)
£80,000–99,999	4 (7)
£100,000–149,999	4 (7)
>£149,999	3 (5.3)

Abbreviation: ICU, intensive care unit.

Mean cognitive composite [mean (*SD*) = 91.5 (13.3); *t*(55) = −4.76, pFWE < 0.0001], language composite [mean (*SD*) = 89.8 (16.6); *t*(52) = −4.45, pFWE < 0.0001], and motor composite [mean (*SD*) = 93.4 (12.8); *t*(54) = −3.84, pFWE = 0.000329] scores were significantly below the Bayley‐III standardized test mean [mean (*SD*) = 100 (15)]. For cognitive composite scores, three children scored below two standard deviations (<70) and 10 scored 1–2 standard deviations (70–84) below the standardized test mean. For language composite scores, four children scored below two standard deviations and 18 scored 1–2 standard deviations below the test mean. For motor composite scores, four scored below two standard deviations and three scored 1–2 standard deviations below the test mean.

Thirty‐five (63.6%) participants had no visible brain injury on presurgical MRI (Table 1). Eleven infants were classified as having a mild brain injury (10 mild WMI, one small cerebellar hemorrhage). Six infants had a moderate brain injury (four moderate WMI, one moderate WMI with cerebellar hemorrhage, one moderate cerebellar hemorrhage). Three children had a severe brain injury (two arterial ischemic infarcts and one severe WMI).

Cognitively stimulating parenting scale scores were significantly correlated with cognition and language abilities (Table 2). There were no other significant associations between clinical and environmental factors and Bayley‐III scores.

**Table 2:** Associations between Bayley‐III scores and clinical and environmental factors

	Cognitive composite	Language composite	Motor composite
Brain injury rating (moderate and severe injury combined)	H^a^=0.733^c^	H = 0.326	H = 0.823
Cyanotic or acyanotic	U^b^ = 199	U = 194.5	U = 215
Cardiac disease category	H = 1.83	H = 1.26	H = 1.04
Days in ICU	Rho = 0.002	Rho = 0.049	Rho = 0.052
Time on bypass	Rho = 0.221	Rho = 0.198	Rho = 0.023
Time to surgery	Rho = 0.071	Rho = 0.073	Rho = 0.051
Maternal education level	H = 3.93	H = 4.34	H = 1.22
Total annual household income (four categories)	H = 4.95	H = 2.76	H = 3.55
Cognitively stimulating parenting scale	Rho = 0.529^d^	Rho = 0.453^e^	Rho = 0.299

^a^ H is Kruskal–Wallis statistic.

^b^ U is Mann–Whitney statistic.

^c^ pFWE = 1.00 unless otherwise indicated.

^d^ pFWE = 0.0017.

^e^ pFWE = 0.033.

**Figure 3:**
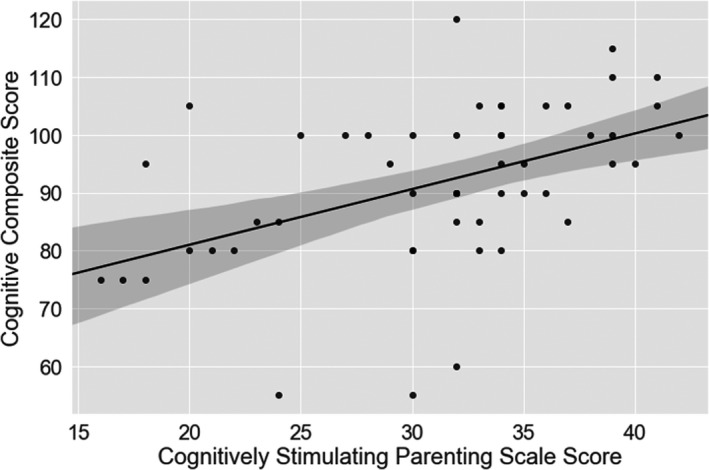
Relationship between cognitively stimulating parenting scale score and cognitive composite scores in toddlers with CHD

Cognitively stimulating parenting scale scores significantly predicted cognitive composite score (Figure 3) when co‐varying for gestational age at birth, sex, and maternal level of education (*F*(6, 40) = 3.04, *p* = 0.015, *R* ^2^ = 0.313, adjusted *R* ^2^ = 0.210; Table 3). The full model explained 30% of the variance in cognitive composite scores. Cognitively stimulating parenting scale scores were not a significant predictor of language composite score when co‐varying for gestational age at birth, sex, and maternal level of education (*F*(6, 38) = 2.21, *p* = 0.064, *R* ^2^ = 0.258, adjusted *R* ^2^ = 0.141; Table 3). A post hoc regression revealed adding brain injury severity rating and categorization of cardiac defect did not alter the relationship between stimulating parenting and cognitive scores (Table 4). Mean cognitive composite scores in infants with highly stimulating parenting were 20 points higher than in infants with the lowest cognitively stimulating parenting scale scores (Appendix S1).

**Table 3:** Regression coefficients predicting cognitive composite score and language composite score in children with CHD.

	Cognitive composite			Language composite		
	Coefficient (*standard error*)	Coefficient 95% confidence intervals	*p*‐Value	Coefficient (*standard error*)	Coefficient 95% confidence intervals	*p*‐Value
Gestational age at birth	1.10 (0.97)	−0.870 to 3.07	0.266	0.499 (1.25)	−2.04 to 3.03	0.693
Male Sex	−0.972 (3.88)	−8.81 to 6.87	0.803	−8.25 (5.05)	−18.5 to 1.97	0.110
A‐levels	3.70 (8.45)	−13.4 to 20.8	0.664	5.00 (10.9)	−17.0 to 27.0	0.648
Vocational education/college	5.76(5.93)	−6.22 to 17.7	0.337	2.20 (7.61)	−13.2 to 17.6	0.774
Higher education	−4.38 (6.33)	−17.2 to 8.41	0.493	5.16 (8.17)	−11.4 to 21.7	0.531
Cognitively stimulating parenting scale	1.19 (0.343)	0.492 to 1.88	0.001	0.791 (0.443)	−0.105 to 1.686	0.082

**Table 4:** Regression coefficients predicting cognitive composite score composite score including additional co‐variates^a^

Variables	Coefficient (standard error)	*p*‐Value	Coefficient 95% confidence intervals
Gestational age at birth	1.17 (1.12)	0.303	−1.11 to 3.45
Male sex	0.266 (4.47)	0.953	−8.82 to 9.36
A‐levels	4.06 (10.1)	0.689	−16.4 to 24.5
Vocational education/college	4.40 (6.92)	0.529	−9.66 to 18.5
Higher education	−6.14 (7.50)	0.419	−21.4 to 9.11
Left heart abnormalities	4.02 (5.35)	0.457	−6.84 to 14.9
Right heart abnormalities	0.448 (4.46)	0.921	−8.61 to 9.51
Mild brain injury	−0.640 (5.12)	0.901	−11.0 to 9.77
Moderate brain injury	3.53 (6.33)	0.581	−9.33 to 16.4
Severe brain injury	−5.30 (8.35)	0.529	−22.2 to 11.6
Cognitively stimulating parenting scale	1.27 (0.369)	0.002	0.516 to 2.02

^a^ *F*(11, 34) = 1.7, *p* = 0.115, *R* ^2^ = 0.355 adjusted *R* ^2^ = 0.147.

## 4 DISCUSSION

This study investigated the influence of environmental, perioperative clinical risk, and disease‐related factors on cognitive, language, and motor abilities at 22 months in children with CHD. A cognitively stimulating home environment was associated with better cognitive abilities, and this relationship was not explained by level of maternal education. Post hoc analyses also revealed type of cardiac defect, and preoperative brain injury severity did not account for the relationship between home environment and cognition. Cardiac physiology, preoperative brain injury severity, and perioperative clinical factors were not associated with outcome scores.

To our knowledge, this is the first study reporting the relationship between a stimulating home environment and cognitive abilities in toddlers with CHD. Extensive research has characterized the relationship between stimulating home environment and improved cognition in children without congenital heart disease, including in low‐ and middle‐income countries, even when controlling for socioeconomic factors (Aboud et al., 2013; Nguyen et al., 2018; Obradović et al., 2016; Pitchik et al., 2018). There is some evidence that home environment can enable children to overcome the effects of socioeconomic status and ill health on early cognitive development (Nampijja et al., 2018; Ronfani et al., 2015). In children born prematurely or with low birthweight, better home environment has been associated with improved cognitive outcome (Bradley et al., 1994; Lynch & Gibbs, 2017; Treyvaud et al., 2012; Wolke et al., 2013) and home environment mediates the relationship between socioeconomic status and cognitive development (Linver et al., 2002). Early intervention programs have also been shown to improve cognitive outcomes throughout early childhood in children born prematurely (Spittle et al., 2015). Providing a stimulating home environment may be a potential interventional strategy to promote cognitive development in children with CHD.

In rodent models of neurological disorders, environmental enrichment paradigms combining exercise, social interaction, as well as novel, complex and stimulating experiences improve performance on cognitive tasks with research pointing to a general mechanism of experience‐dependent neural plasticity (Nithianantharajah & Hannan, 2006). To our knowledge, there have been no studies examining the effect of environmental enrichment in a rodent model of CHD; however, rodents exposed to neonatal hypoxia‐ischemia, an event which deprives the brain of glucose and oxygen, show better motor and behavioral development when exposed to an enriched environment with opportunities for investigation, peer interaction, and exercise compared to a standard laboratory environment (Galeano et al., 2015; Kiss et al., 2013; Rojas et al., 2015). These behavioral improvements may be accompanied by attenuated brain volume loss and reduced histological tissue damage (Durán‐Carabali et al., 2018, 2019; Schuch et al., 2016). In a mouse model of preterm brain injury, prolonged early environmental enrichment was associated with improved oligodendrogenesis, myelination, and functional outcomes (Forbes et al., 2020). Interestingly, the authors report that environmental enrichment did not promote myelination in healthy tissue, suggesting at‐risk populations may uniquely benefit from environmental interventions. Further animal research is needed to determine whether an enriched environment might modify the detrimental effects of CHD on brain and cognitive development; however, we hypothesize that this effect may underlie the relationship between cognition and stimulating parenting reported in this study.

We also reported that cognitive, motor, and language scores were significantly lower than the standardized population mean at 22 months in children with CHD. This is in line with previous literature which report a small (<1*SD*) but significant decrease in cognitive, motor, and language scores on the Bayley Scales of Infant Development in young children with CHD without syndromic diagnoses (Gaynor et al., 2015; Gunn et al., 2016; Latal, 2016; Mebius et al., 2017; Wray, 2006). It confirms that this group of infants with adverse outcomes are susceptible to environmental influences.

In previous studies, longer times to surgery in infants with hypoplastic left heart syndrome have been associated with increased morbidity, healthcare costs, and postsurgical white matter injury (Anderson et al., 2015; Lynch et al., 2014). Longer hospital stay post‐surgery has been associated with lower cognitive scores in samples of children with hypoplastic left heart syndrome and other single ventricle physiologies, transposition of the great arteries, and tetralogy of Fallot (Gaynor et al., 2014, 2016; Hansen et al., 2016). Our study included a wider range of CHD diagnoses than those previously published, which may explain why our findings with respect to increased time to surgery and longer hospital stay differ to previous reports. The size of the study group may also explain these missing associations, but equally suggests that the home environment has a powerful effect on cognitive development. It is also possible that clinical variables differentially influence developmental outcome in different types of CHD; however, larger samples are needed to address this hypothesis.

We found no relationship between preoperative brain injury severity and outcome at 22 months. The literature regarding preoperative brain injury and developmental outcome is inconsistent (Mebius et al., 2017). Claessens and colleagues reported moderate‐severe white matter injury perioperatively was associated with poorer cognitive outcome and injury to the descending motor tracts in the posterior limb of the internal capsule was associated with poorer motor outcome (Claessens et al., 2018). However, the incidence of brain injury in our group was low, with almost two‐thirds having no evidence of injury and only three having severe injury. In children born prematurely, socioeconomic status may attenuate the effect of brain injury on cognitive development (Benavente‐Fernández et al., 2019). Environmental factors such as a stimulating home environment may attenuate the impact of brain injury on cognitive outcome in other at‐risk groups; however, this requires further research.

### 4.1 Limitations and future research

Our study has some limitations. The sample size is relatively small, and future studies with larger samples are required to confirm the relationship between home environment and outcome in CHD. In addition, it is possible that postsurgical brain injury, which we did not assess, has a larger influence on subsequent outcome. The cognitively stimulating parenting scale, while derived from validated tests in a principled fashion, has been used in only a few studies (Wolke et al., 2013) and confirmation of these results is required. Our study considered the effect of cognitively stimulating parenting on outcome, yet parenting behaviors encompass other characteristics known to affect development such as sensitivity (Wolke et al., 2013), responsiveness (Coscia et al., 2001; Lynch & Gibbs, 2017), and family functioning (Downes et al., 2019). Future research could characterize the effect of other aspects of parenting on development in children with CHD. In addition, there is limited evidence from randomized controlled trials that parenting interventions can improve cognitive outcome in early childhood (Bonnier, 2008; Shah et al., 2016) and therefore randomized trials are required to assess whether interventions to promote a stimulating home environment improves cognitive outcome in children with CHD.

Finally, in this study we considered the effect of home environment at 22 months on developmental outcome in CHD. Prenatal as well as postnatal environmental enrichment improves outcome in rats with perinatal hypoxia‐ischemia (Duran‐Carabali et al., 2018; Durán‐Carabali et al., 2019), and we have previously observed altered white matter development in infants born prematurely who experienced prenatal stress exposure (Lautarescu et al., 2019). An improved prenatal environment such as increased maternal exercise or reduced maternal stress may improve brain and cognitive development (Miguel et al., 2019). The effect of prenatal maternal well‐being on development in infants with CHD requires further study.

## 5 CONCLUSIONS

A stimulating home environment is associated with higher cognitive scores at 22 months in infants with CHD. This provides the first evidence of a modifiable environmental factor in children with CHD who are at risk for intellectual impairments and other learning disabilities. Supporting parents to provide a stimulating home environment in early childhood may support early cognitive development in this population.

## Supporting information

Appendix S1

## Data Availability

The data that support the findings of this study are available from the corresponding author upon reasonable request.

## References

[infa12376-bib-0001] Aboud, F. E. , Singla, D. R. , Nahil, M. I. , & Borisova, I. (2013). Effectiveness of a parenting program in Bangladesh to address early childhood health, growth and development. Social Science and Medicine, 97, 250–258. 10.1016/j.socscimed.2013.06.020 23871435

[infa12376-bib-0002] Anderson, B. R. , Ciarleglio, A. J. , Salavitabar, A. , Torres, A. , & Bacha, E. A. (2015). Earlier stage 1 palliation is associated with better clinical outcomes and lower costs for neonates with hypoplastic left heart syndrome. The Journal of Thoracic and Cardiovascular Surgery, 149(1), 205–210.e1. 10.1016/j.jtcvs.2014.07.094 25227701

[infa12376-bib-0003] Bayley, N. (2006). Bayley scales of infant and toddler development (3rd ed.). PsychCorp, Pearson.

[infa12376-bib-0004] Beca, J. , Gunn, J. K. , Coleman, L. , Hope, A. , Reed, P. W. , Hunt, R. W. , Finucane, K. , Brizard, C. , Dance, B. , & Shekerdemian, L. S. (2013). New white matter brain injury after infant heart surgery is associated with diagnostic group and the use of circulatory arrest. Circulation, 127(9), 971–979. 10.1161/CIRCULATIONAHA.112.001089 23371931

[infa12376-bib-0005] Benavente‐Fernández, I. , Synnes, A. , Grunau, R. E. , Chau, V. , Ramraj, C. , Glass, T. , Cayam‐Rand, D. , Siddiqi, A. , & Miller, S. P. (2019). Association of socioeconomic status and brain injury with neurodevelopmental outcomes of very preterm children. JAMA Network Open, 2(5), e192914 10.1001/jamanetworkopen.2019.2914 31050776 PMC6503490

[infa12376-bib-0006] Bonnier, C. (2008). Evaluation of early stimulation programs for enhancing brain development. Acta Paediatrica, 97(7), 853–858. 10.1111/j.1651-2227.2008.00834.x 18482172

[infa12376-bib-0007] Bradley, R. , & Caldwell, B. (1984). Home observation for measurement of the environment.

[infa12376-bib-0008] Bradley, R. H. , & Corwyn, R. F. (2002). Socioeconomic status and child development. Annual Review of Psychology, 53, 371–399. 10.1146/annurev.psych.53.100901.135233 11752490

[infa12376-bib-0009] Bradley, R. H. , Whiteside, L. , Mundfrom, D. J. , Casey, P. H. , Kelleher, K. J. , & Pope, S. K. (1994). Early indications of resilience and their relation to experiences in the home environments of low birthweight, premature children living in poverty. Child Development, 65(2), 346–360. 8013226

[infa12376-bib-0010] Claessens, N. H. P. , Algra, S. O. , Ouwehand, T. L. , Jansen, N. J. G. , Schappin, R. , Haas, F. , Eijsermans, M. J. C. , Vries, L. S. , Benders, M. J. N. L. , Moeskops, P. , Išgum, I. , Haastert, I. C. , Groenendaal, F. , & Breur, J. M. P. J. (2018). Perioperative neonatal brain injury is associated with worse school‐age neurodevelopment in children with critical congenital heart disease. Developmental Medicine and Child Neurology, 60(10), 1052–1058. 10.1111/dmcn.13747 29572821

[infa12376-bib-0011] Coscia, J. M. , Christensen, B. K. , Henry, R. R. , Wallston, K. , Radcliffe, J. , & Rutstein, R. (2001). Effects of Home environment, socioeconomic status, and health status on cognitive functioning in children with HIV‐1 infection. Journal of Pediatric Psychology, 26(6), 321–329. 10.1093/jpepsy/26.6.321 11490033

[infa12376-bib-0012] Costello, J. M. , Polito, A. , Brown, D. W. , McElrath, T. F. , Graham, D. A. , Thiagarajan, R. R. , Bacha, E. A. , Allan, C. K. , Cohen, J. N. , & Laussen, P. C. (2010). Birth before 39 weeks gestation is associated with worse outcomes in neonates with heart disease. Pediatrics, 126(2), 277–284. 10.1542/peds.2009-3640 20603261

[infa12376-bib-0013] Downes, M. , de Haan, M. , Telfer, P. T. , & Kirkham, F. J. (2019). The role of family functioning in the development of executive functions in preschool children with sickle cell anemia. Developmental Neuropsychology, 44(6), 452–467. 10.1080/87565641.2019.1660779 31450996

[infa12376-bib-0014] Durán‐Carabali, L. E. , Arcego, D. M. , Odorcyk, F. K. , Reichert, L. , Cordeiro, J. L. , Sanches, E. F. , Freitas, L. D. , Dalmaz, C. , Pagnussat, A. , & Netto, C. A. (2018). Prenatal and early postnatal environmental enrichment reduce acute cell death and prevent neurodevelopment and memory impairments in rats submitted to neonatal hypoxia ischemia. Molecular Neurobiology, 55(5), 3627–3641. 10.1007/s12035-017-0604-5 28523564

[infa12376-bib-0015] Durán‐Carabali, L. E. , Arcego, D. M. , Sanches, E. F. , Odorcyk, F. K. , Marques, M. R. , Tosta, A. , Reichert, L. , Carvalho, A. S. , Dalmaz, C. , & Netto, C. A. (2019). Preventive and therapeutic effects of environmental enrichment in Wistar rats submitted to neonatal hypoxia‐ischemia. Behavioural Brain Research, 359, 485–497. 10.1016/j.bbr.2018.11.036 30496770

[infa12376-bib-0016] EUROCAT (2015). Eurocat prevalence tables. http://www.eurocat‐network.eu/accessprevalencedata/prevalencetables

[infa12376-bib-0017] Ewer, A. K. , Middleton, L. J. , Furmston, A. T. , Bhoyar, A. , Daniels, J. P. , Thangaratinam, S. , Deeks, J. J. , & Khan, K. S. (2011). Pulse oximetry screening for congenital heart defects in newborn infants (PulseOx): A test accuracy study. The Lancet, 378(9793), 785–794. 10.1016/S0140-6736(11)60753-8 21820732

[infa12376-bib-0018] Forbes, T. A. , Goldstein, E. Z. , Dupree, J. L. , Jablonska, B. , Scafidi, J. , Adams, K. L. , Imamura, Y. , Hashimoto‐Torii, K. , & Gallo, V. (2020). Environmental enrichment ameliorates perinatal brain injury and promotes functional white matter recovery. Nature Communications, 11(1), 964 10.1038/s41467-020-14762-7 PMC7031237 32075970

[infa12376-bib-0019] Galeano, P. , Blanco, E. , Logica Tornatore, T. M. A. , Romero, J. I. , Holubiec, M. I. , Rodríguez de Fonseca, F. , & Capani, F. (2015). Life‐long environmental enrichment counteracts spatial learning, reference and working memory deficits in middle‐aged rats subjected to perinatal asphyxia. Frontiers in Behavioral Neuroscience, 8, 406 10.3389/fnbeh.2014.00406 25601829 PMC4283640

[infa12376-bib-0020] Gaynor, J. W. , Ittenbach, R. F. , Gerdes, M. , Bernbaum, J. , Clancy, R. R. , McDonald‐McGinn, D. M. , Zackai, E. H. , Wernovsky, G. , Nicolson, S. C. , & Spray, T. L. (2014). Neurodevelopmental outcomes in preschool survivors of the Fontan procedure. The Journal of Thoracic and Cardiovascular Surgery, 147(4), 1276–1283.e5. 10.1016/j.jtcvs.2013.12.019 24521968 PMC5662937

[infa12376-bib-0021] Gaynor, J. W. , Stopp, C. , Wypij, D. , Andropoulos, D. B. , Atallah, J. , Atz, A. M. , Beca, J. , Donofrio, M. T. , Duncan, K. , Ghanayem, N. S. , Goldberg, C. S. , Hövels‐Gürich, H. , Ichida, F. , Jacobs, J. P. , Justo, R. , Latal, B. , Li, J. S. , Mahle, W. T. , McQuillen, P. S. , … Newburger, J. W. (2016). Impact of operative and postoperative factors on neurodevelopmental outcomes after cardiac operations. The Annals of Thoracic Surgery, 102(3), 843–849. 10.1016/j.athoracsur.2016.05.081 27496628

[infa12376-bib-0022] Gaynor, J. W. , Stopp, C. , Wypij, D. , Andropoulos, D. B. , Atallah, J. , Atz, A. M. , Beca, J. , Donofrio, M. T. , Duncan, K. , Ghanayem, N. S. , Goldberg, C. S. , Hövels‐Gürich, H. , Ichida, F. , Jacobs, J. P. , Justo, R. , Latal, B. , Li, J. S. , Mahle, W. T. , McQuillen, P. S. , … Newburger, J. W. (2015). Neurodevelopmental outcomes after cardiac surgery in infancy. Pediatrics, 135(5), 816–825. 10.1542/peds.2014-3825 25917996 PMC4533222

[infa12376-bib-0023] Gerstle, M. , Beebe, D. W. , Drotar, D. , Cassedy, A. , & Marino, B. S. (2016). Executive functioning and school performance among pediatric survivors of complex congenital heart disease. The Journal of Pediatrics, 173, 154–159. 10.1016/j.jpeds.2016.01.028 26875011 PMC4884495

[infa12376-bib-0024] Gunn, J. K. , Beca, J. , Hunt, R. W. , Goldsworthy, M. , Brizard, C. P. , Finucane, K. , Donath, S. , & Shekerdemian, L. S. (2016). Perioperative risk factors for impaired neurodevelopment after cardiac surgery in early infancy. Archives of Disease in Childhood, 101(11), 1010–1016. 10.1136/archdischild-2015-309449 27272973

[infa12376-bib-0025] Hansen, J. H. , Rotermann, I. , Logoteta, J. , Jung, O. , Dütschke, P. , Scheewe, J. , & Kramer, H.‐H. (2016). Neurodevelopmental outcome in hypoplastic left heart syndrome: Impact of perioperative cerebral tissue oxygenation of the Norwood procedure. The Journal of Thoracic and Cardiovascular Surgery, 151(5), 1358–1366. 10.1016/j.jtcvs.2016.02.035 27085616

[infa12376-bib-0026] Hoffman, J. I. E. , & Kaplan, S. (2002). The incidence of congenital heart disease. Journal of the American College of Cardiology, 39(12), 1890–1900. 10.1016/s0735-1097(02)01886-7 12084585

[infa12376-bib-0027] Kelly, C. J. , Arulkumaran, S. , Tristão Pereira, C. , Cordero‐Grande, L. , Hughes, E. J. , Teixeira, R. P. A. G. , Steinweg, J. K. , Victor, S. , Pushparajah, K. , Hajnal, J. V. , Simpson, J. , Edwards, A. D. , Rutherford, M. A. , & Counsell, S. J. (2019). Neuroimaging findings in newborns with congenital heart disease prior to surgery: An observational study. Archives of Disease in Childhood, 104(11), 1042–1048. 10.1136/archdischild-2018-314822 31243012 PMC6801127

[infa12376-bib-0028] Kiss, P. , Vadasz, G. , Kiss‐Illes, B. , Horvath, G. , Tamas, A. , Reglodi, D. , & Koppan, M. (2013). Environmental enrichment decreases asphyxia‐induced neurobehavioral developmental delay in neonatal rats. International Journal of Molecular Sciences, 14(11), 22258–22273. 10.3390/ijms141122258 24232451 PMC3856064

[infa12376-bib-0029] Latal, B. (2016). Neurodevelopmental outcomes of the child with congenital heart disease. Clinics in Perinatology, 43(1), 173–185. 10.1016/j.clp.2015.11.012 26876129

[infa12376-bib-0030] Lautarescu, A. , Pecheva, D. , Nosarti, C. , Nihouarn, J. , Zhang, H. , Victor, S. , Craig, M. , Edwards, A. D. , & Counsell, S. J. (2019). Maternal prenatal stress is associated with altered uncinate fasciculus microstructure in premature neonates. Biological Psychiatry, 87(6), 559–569. 10.1016/j.biopsych.2019.08.010 31604519 PMC7016501

[infa12376-bib-0031] Linver, M. R. , Brooks‐Gunn, J. , & Kohen, D. E. (2002). Family processes as pathways from income to young children’s development. Developmental Psychology, 38(5), 719–734. 12220050

[infa12376-bib-0032] Lynch, J. M. , Buckley, E. M. , Schwab, P. J. , McCarthy, A. L. , Winters, M. E. , Busch, D. R. , Xiao, R. , Goff, D. A. , Nicolson, S. C. , Montenegro, L. M. , Fuller, S. , Gaynor, J. W. , Spray, T. L. , Yodh, A. G. , Naim, M. Y. , & Licht, D. J. (2014). Time to surgery and preoperative cerebral hemodynamics predict postoperative white matter injury in neonates with hypoplastic left heart syndrome. The Journal of Thoracic and Cardiovascular Surgery, 148(5), 2181–2188. 10.1016/j.jtcvs.2014.05.081 25109755 PMC4254035

[infa12376-bib-0033] Lynch, J. L. , & Gibbs, B. G. (2017). Birth weight and early cognitive skills: Can parenting offset the link? Maternal and Child Health Journal, 21(1), 156–167. 10.1007/s10995-016-2104-z 27469110

[infa12376-bib-0034] Marino, B. S. , Lipkin, P. H. , Newburger, J. W. , Peacock, G. , Gerdes, M. , Gaynor, J. W. , Mussatto, K. A. , Uzark, K. , Goldberg, C. S. , Johnson, W. H. J. , Li, J. , Smith, S. E. , Bellinger, D. C. , & Mahle, W. T. (2012). Neurodevelopmental outcomes in children with congenital heart disease: Evaluation and management: A scientific statement from the American Heart Association. Circulation, 126(9), 1143–1172. 10.1161/CIR.0b013e318265ee8a 22851541

[infa12376-bib-0035] Mebius, M. J. , Kooi, E. M. W. , Bilardo, C. M. , & Bos, A. F. (2017). Brain injury and neurodevelopmental outcome in congenital heart disease: A systematic review. Pediatrics, 140(1), e20164055 10.1542/peds.2016-4055 28607205

[infa12376-bib-0036] Miguel, P. M. , Pereira, L. O. , Silveira, P. P. , & Meaney, M. J. (2019). Early environmental influences on the development of children’s brain structure and function. Developmental Medicine and Child Neurology, 61(10), 1127–1133. 10.1111/dmcn.14182 30740660

[infa12376-bib-0037] Nampijja, M. , Kizindo, R. , Apule, B. , Lule, S. , Muhangi, L. , Titman, A. , Elliott, A. , Alcock, K. , & Lewis, C. (2018). The role of the home environment in neurocognitive development of children living in extreme poverty and with frequent illnesses: A cross‐sectional study. Wellcome Open Research, 3, 152 10.12688/wellcomeopenres.14702.1 30687794 PMC6338129

[infa12376-bib-0038] Nguyen, P. H. , DiGirolamo, A. M. , Gonzalez‐Casanova, I. , Young, M. , Kim, N. , Nguyen, S. , Martorell, R. , & Ramakrishnan, U. (2018). Influences of early child nutritional status and home learning environment on child development in Vietnam. Maternal & Child Nutrition, 14(1), e12468 10.1111/mcn.12468 PMC6865959 28585371

[infa12376-bib-0039] Nithianantharajah, J. , & Hannan, A. J. (2006). Enriched environments, experience‐dependent plasticity and disorders of the nervous system. Nature Reviews Neuroscience, 7(9), 697–709. 10.1038/nrn1970 16924259

[infa12376-bib-0040] Obradović, J. , Yousafzai, A. , Finch, J. , & Rasheed, M. (2016). Maternal scaffolding and home stimulation: Key mediators of early intervention effects on children’s cognitive development. Developmental Psychology, 52, 10.1037/dev0000182 27505702

[infa12376-bib-0041] Pitchik, H. O. , Fawzi, W. W. , McCoy, D. C. , Darling, A. M. , Abioye, A. I. , Tesha, F. , Smith, E. R. , Mugusi, F. , & Sudfeld, C. R. (2018). Prenatal nutrition, stimulation, and exposure to punishment are associated with early child motor, cognitive, language, and socioemotional development in Dar es Salaam, Tanzania. Child: Care, Health and Development, 44(6), 841–849. 10.1111/cch.12605 PMC8272522 30124230

[infa12376-bib-0042] Rojas, J. J. , Deniz, B. F. , Schuch, C. P. , Carletti, J. V. , Deckmann, I. , Diaz, R. , Matte, C. , dos Santos, T. M. , Wyse, A. T. , Netto, C. A. , & Pereira, L. O. (2015). Environmental stimulation improves performance in the ox‐maze task and recovers Na+, K+‐ATPase activity in the hippocampus of hypoxic‐ischemic rats. Neuroscience, 291, 118–127. 10.1016/j.neuroscience.2015.01.017 25617656

[infa12376-bib-0043] Ronfani, L. , Vecchi Brumatti, L. , Mariuz, M. , Tognin, V. , Bin, M. , Ferluga, V. , Knowles, A. , Montico, M. , & Barbone, F. (2015). The complex interaction between home environment, socioeconomic status, maternal iq and early child neurocognitive development: A multivariate analysis of data collected in a newborn cohort study. PLoS One, 10(5), e0127052 10.1371/journal.pone.0127052 25996934 PMC4440732

[infa12376-bib-0044] Sarrechia, I. , Miatton, M. , De Wolf, D. , Francois, K. , Gewillig, M. , Meyns, B. , & Vingerhoets, G. (2016). Neurocognitive development and behaviour in school‐aged children after surgery for univentricular or biventricular congenital heart disease. European Journal of Cardio‐Thoracic Surgery, 49(1), 167–174. 10.1093/ejcts/ezv029 25694470

[infa12376-bib-0045] Schuch, C. P. , Diaz, R. , Deckmann, I. , Rojas, J. J. , Deniz, B. F. , & Pereira, L. O. (2016). Early environmental enrichment affects neurobehavioral development and prevents brain damage in rats submitted to neonatal hypoxia‐ischemia. Neuroscience Letters, 617, 101–107. 10.1016/j.neulet.2016.02.015 26872850

[infa12376-bib-0046] Shah, R. , Kennedy, S. , Clark, M. D. , Bauer, S. C. , & Schwartz, A. (2016). Primary care‐based interventions to promote positive parenting behaviors: A meta‐analysis. Pediatrics, 137(5), e20153393 10.1542/peds.2015-3393 27244800 PMC4845871

[infa12376-bib-0047] Spittle, A. , Orton, J. , Anderson, P. J. , Boyd, R. , & Doyle, L. W. (2015). Early developmental intervention programmes provided post hospital discharge to prevent motor and cognitive impairment in preterm infants. The Ochrane Database of Systematic Reviews, 11, CD005495 10.1002/14651858.CD005495.pub4 PMC8612699 26597166

[infa12376-bib-0048] Treyvaud, K. , Inder, T. E. , Lee, K. J. , Northam, E. A. , Doyle, L. W. , & Anderson, P. J. (2012). Can the home environment promote resilience for children born very preterm in the context of social and medical risk? Journal of Experimental Child Psychology, 112(3), 326–337. 10.1016/j.jecp.2012.02.009 22480454

[infa12376-bib-0049] Wolke, D. , Jaekel, J. , Hall, J. , & Baumann, N. (2013). Effects of Sensitive Parenting on the Academic Resilience of Very Preterm and Very Low Birth Weight Adolescents. Journal of Adolescent Health, 53(5), 642–647. 10.1016/j.jadohealth.2013.06.014 23910570

[infa12376-bib-0050] Wray, J. O. (2006). Intellectual development of infants, children and adolescents with congenital heart disease. Developmental Science, 9(4), 368–378. 10.1111/j.1467-7687.2006.00502.x 16764610

[infa12376-bib-0051] Wray, J. , & Sensky, T. (2001). Congenital heart disease and cardiac surgery in childhood: Effects on cognitive function and academic ability. Heart, 85(6), 687–691. 10.1136/heart.85.6.687 11359753 PMC1729789

[infa12376-bib-0052] Wren, C. , & Sullivan, J. J. (2001). Survival with congenital heart disease and need for follow up in adult life. Heart, 85(4), 438–443. 10.1136/heart.85.4.438 11250973 PMC1729699

[infa12376-bib-0053] Zhou, S. J. , Baghurst, P. , Gibson, R. A. , & Makrides, M. (2007). Home environment, not duration of breast‐feeding, predicts intelligence quotient of children at four years. Nutrition, 23(3), 236–241. 10.1016/j.nut.2006.12.011 17320351

