# Supplementary material for: Cognitive function in toddlers with congenital heart disease: The impact of a stimulating home environment
Source: Infancy. 2020 Nov 19;26(1):184–99. doi: 10.1111/infa.12376 (PMC7894304; doi:10.1111/infa.12376)

Appendix S1

We split the data into quintiles according to score on the Cognitively Stimulating Parenting Scale (CSPS) and examined cognitive composite scores of the top and bottom 20% of the sample. Cognitive Composite Scores from infants who were in the top 20% (CSPS score 37-42; N=11) on the CSPS were all within the normal range (≥85; range 85-115). The mean (SD) cognitive score was 101.8 (8.45); 10 points higher than the full sample mean of 91.5 (13.3). In comparison the toddlers with the bottom 20% of parenting scores (CSPS score 16-24, N=11) had a mean (SD) Cognitive Composite Score of 80.9 (12.6) (Range 55-105). Seven infants scored more than one standard deviation below the test mean (<85) (See Figure).


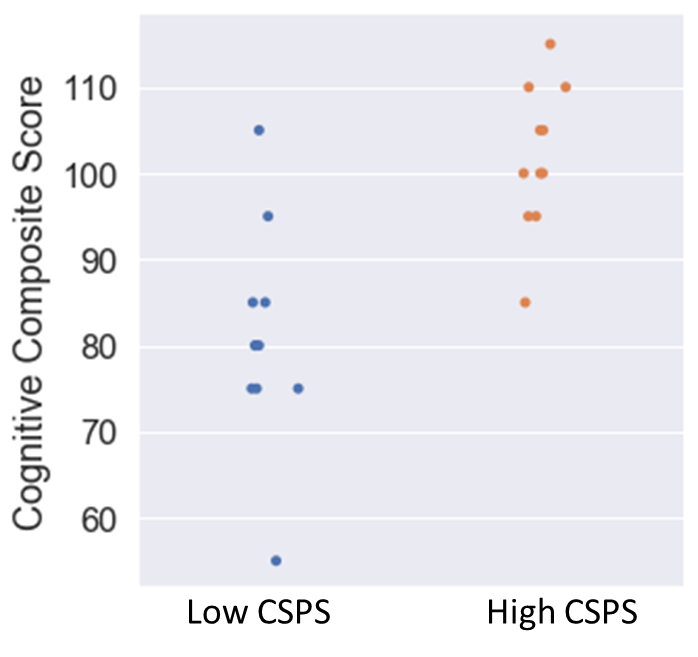

Supplement: Supplementary file 1 — Appendix S1 [file INFA-26-184-s001.docx]
